# SMACH: A Rare, Non-Progressive Retinal Disorder – Key Insights into Its Clinical and Imaging Characteristics. A Case Report

**DOI:** 10.22336/rjo.2026.44

**Published:** 2026

**Authors:** Mauricio Arango-Hurtado, Karol Quintero-Lizcano, Javier Andres Díaz-Vargas, Sara Turizo Mejía, Sarita Restrepo Velásquez

**Affiliations:** 1 Department of Ophthalmology, Faculty of Medicine, CES University, Medellín, Colombia; 2 Retina and Vitreous Department, Clofán Clinic, Medellín, Colombia

**Keywords:** Stellate Multiform Amelanotic Choroidopathy (SMACH), subretinal fluid, multimodal imaging, emerging diseases, SMACH = Stellate Multiform Amelanotic Choroidopathy, RPE = retinal pigment epithelium, OCT = Optical Coherence Tomography, OCT-A = Optical Coherence Tomography Angiography, FA = fluorescein angiography, SRF = subretinal fluid, BCVA = Best-corrected visual acuity, OS = left eye, OD = right eye

## Abstract

**Objective:**

To report a case of a Stellate Multiform Amelanotic Choroidopathy (SMACH). A newly recognized, rare retinal disorder that remains largely underreported in the literature.

**Methods:**

Case report and review of literature.

**Results:**

We present the case of a 67-year-old asymptomatic woman with a nasal parafoveal hypopigmented lesion. Multimodal imaging, including Optical Coherence Tomography, Optical Coherence Tomography Angiography, and fluorescein angiography, revealed hyperreflective choroidal thickening, minimal subretinal fluid, and a digitiform pattern, with no signs of neovascularization.

**Discussion:**

SMACH is a rare, benign choroidal disorder whose variable clinical presentation can complicate accurate diagnosis. Understanding its non-progressive natural history is critical for differentiating it from vision-threatening pathologies, such as choroidal tumors, macular neovascularization, or pachychoroid spectrum diseases, reducing the risk of misdiagnosis and unnecessary therapeutic intervention.

**Conclusions:**

This case enhances our understanding of SMACH and underscores the value of multimodal imaging in identifying this rare condition, providing key insights into its diagnostic approach and natural course.

## Introduction

Stellate Multiform Amelanotic Choroidopathy (SMACH) is an emerging and poorly understood retinal condition, with only a limited number of cases reported to date. First described by Van Dijk et al. in 2021, SMACH is recognized as a benign, non-progressive disorder primarily affecting the choroidal-retinal pigment epithelium (RPE) complex. It presents with focal choroidal thickening and hyperreflective changes in the inner choroid, without associated macular neovascularization [1]. Although subretinal fluid (SRF) was initially considered a key marker of SMACH, some reports suggest that up to 40% of cases lack SRF, underscoring the need to identify additional structural features to aid diagnosis. The role of multimodal imaging in better characterizing this condition has been crucial; however, despite advances in imaging techniques, the number of documented cases remains insufficient to understand its pathophysiology and clinical course fully. This report presents a clinical case of SMACH, detailing the patient’s clinical features and imaging findings that contribute to the growing body of knowledge on this rare pathology.

By sharing this case, we aim to provide further evidence of SMACH’s characteristic imaging patterns and underscore the importance of an integrated diagnostic approach for this uncommon retinal disorder.

## Case report

**Fig. 1:**
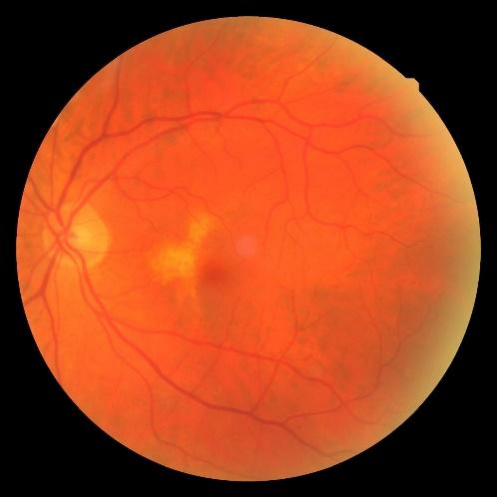
Color fundus photograph of the OS revealed a hypopigmented lesion with digitiform-like extensions in the nasal parafoveal and perifoveal region

A 67-year-old asymptomatic woman with no significant medical history was referred to the retina service due to suspected choroidal osteoma. The patient exhibited an unremarkable anterior segment examination, and her intraocular pressure was within normal limits. Best-corrected visual acuity (BCVA) was 20/25 in both eyes, and the patient reported no visual disturbances or ocular discomfort. Fundus examination revealed a subtle hypopigmented lesion in the nasal parafoveal region of the left eye (OS), extending towards the superior area and the papillomacular bundle (Fig. 1). The right eye (OD) appeared unremarkable, with no visible alterations. Given the unusual presentation, the patient was referred for multimodal imaging to characterize the lesion better and rule out other potential causes, including choroidal tumors, macular neovascularization, or pachychoroid spectrum diseases.

**Fig. 2:**
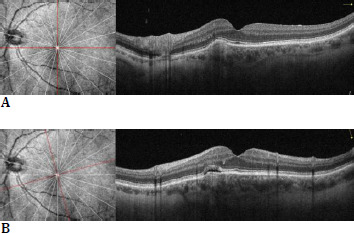
Horizontal (A) and oblique (B) B-scan OCT of the OS with fibrotic changes in the inner choroid and anterior protrusion of the RPE associated with SRF and elongation of the photoreceptors at the site of the lesion

Multimodal imaging provided a detailed view of the lesion’s structural features. Optical Coherence Tomography (OCT) of the left eye revealed hyperreflective choroidal thickening adjacent to the fovea, along with protrusion of the RPE-Bruch’s complex. Notably, minimal SRF was present, a feature commonly associated with SMACH, though in this case, it was not a prominent finding. An irregularity in the outer retina was observed, with elongation of the photoreceptors at the site of the lesion (Fig. 2A, B). These findings raised suspicion for SMACH and prompted further imaging to ensure a comprehensive evaluation.

**Fig. 3:**
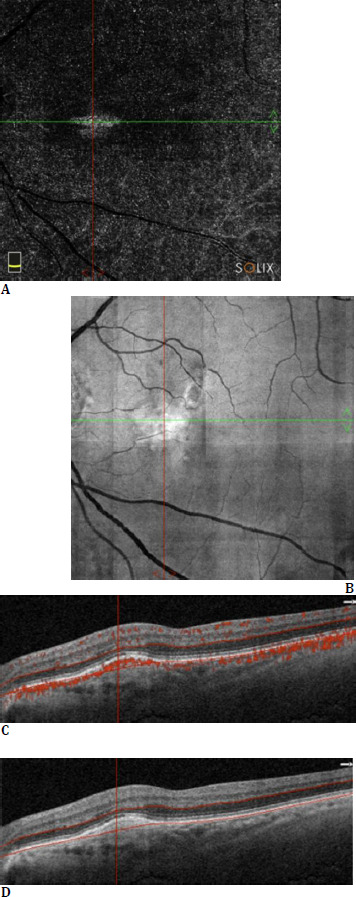
OS imaging. Outer retina angiogram with an irregular hyperreflective parafoveal lesion (A). En face outer retina imaging shows a hyperreflective round lesion with digitiform-like extensions in the nasal parafoveal and perifoveal area (B). Flow overlay confirms normal choroidal flow even in the inner choroid fibrotic lesion (C). Horizontal B-scan OCT confirming segmentation (D)

**Fig. 4:**
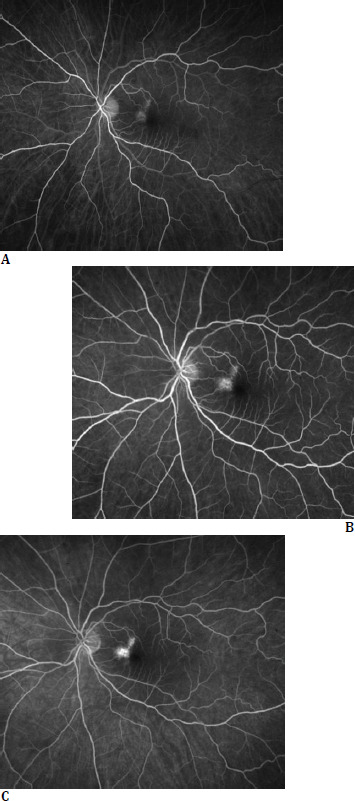
Fluorescein angiography of the OS showed a hyperfluorescent lesion in the nasal parafoveal region, with progressive intensification of fluorescence in the later stages (Early arteriovenous (A), intermediate arteriovenous (B), and late arteriovenous phases (C))

Optical Coherence Tomography Angiography (OCT-A) showed no evidence of macular neovascularization, which is essential for distinguishing SMACH from other conditions that may present with SRF and retinal changes. Additionally, en face OCT imaging segmented at the level of the choroidal lesion revealed hyperreflective changes with distinctive fingerlike projections oriented radially in a stellate configuration, characteristic of SMACH (Fig. 3A-D). Fluorescein angiography (FA) showed a hyperfluorescent lesion in the nasal parafoveal region, with progressive intensification of fluorescence in the later stages, further supporting the diagnosis of SMACH over other potential causes (Fig. 4A-C).

At the 1-year follow-up, the patient’s visual acuity remained stable at 20/25, and no significant changes were observed in the lesion’s appearance. There was no increase in SRF, and the hyperreflective choroidal thickening remained unchanged. These findings are consistent with the non-progressive nature of SMACH, where lesions typically stabilize without significant deterioration in visual function. The patient was advised to continue regular monitoring to track any future changes.

## Discussion

SMACH remains an emergent retinal condition, with only a few documented cases. Although the exact pathophysiology is still unclear, it is thought to involve changes in the choroidal circulation, with fluctuations in hydrostatic pressure contributing to the accumulation of SRF [2]. Despite the presence of SRF in many cases, visual acuity is often preserved, possibly due to compensatory mechanisms within the retina, such as Müller cell activity that maintains the visual cycle.

Recent studies by Ramtohul et al. [3] have established OCT as the gold standard for diagnosing SMACH. Their research highlighted key characteristics, such as hyperreflective fibrotic changes in the inner choroid and anterior protrusion of the RPE complex, which are critical diagnostic features. Furthermore, en face imaging revealed a digitiform pattern, a hallmark feature of SMACH. Notably, SRF was present in only 59% of cases, indicating that SRF is not always a consistent or mandatory diagnostic feature. This underscores the need to recognize other structural and functional characteristics to support the diagnosis.

In this context, multimodal imaging has proven indispensable for characterizing SMACH. The combination of OCT, OCT-A, and fluorescein angiography provides a comprehensive assessment, helping to distinguish SMACH from other retinal pathologies. By integrating these imaging modalities, clinicians can gain a clearer understanding of the unique structural changes associated with SMACH, reducing the risk of misdiagnosis and ensuring more accurate management of this disorder.

Given its non-progressive and benign nature, SMACH can be differentiated from other conditions, such as choroidal tumors, macular neovascularization, and pachychoroid spectrum diseases. Recognition of the specific imaging features of SMACH is critical in preventing misdiagnoses and unnecessary interventions [4,5].

## Conclusion

SMACH is a recently recognized and rare retinal disorder that challenges traditional diagnostic approaches. This case highlights the importance of multimodal imaging in the key features of SMACH. It not only reinforces the characteristic imaging findings but also provides valuable insights into the potential disease course in an individual who remains asymptomatic and stable over an extended follow-up period. The multimodal imaging approach facilitated a comprehensive understanding of the lesion’s structure and confirmed the diagnosis, while also underscoring the importance of continuous monitoring in managing this rare retinal condition.
